# Maternal genetic polymorphisms in the major mitotic checkpoint genes *MAD1L1* and *MAD2L1* associated with the risk of survival in abnormal chromosomal fetuses

**DOI:** 10.3389/fgene.2023.1105184

**Published:** 2023-03-16

**Authors:** Ying Chan, Yize Liu, Yamin Kong, Weiming Xu, Xiaohong Zeng, Haichun Li, Yan Guo, Xinhua Tang, Jinman Zhang, Baosheng Zhu

**Affiliations:** ^1^ Department of Medical Genetics, NHC Key Laboratory of Preconception Health Birth in Western China, Yunnan Provincial Key Laboratory for Birth Defects and Genetic Diseases, First People’s Hospital of Yunnan Province, Affiliated Hospital of Kunming University of Science and Technology, Kunming, China; ^2^ School of Medicine, Kunming University of Science and Technology, Kunming, Yunnan, China

**Keywords:** *MAD1L1*, *MAD2L1*, homocysteine(HYC), fetal chromosome abnormality, polymorphism

## Abstract

**Background:** The genetic etiology of fetal chromosome abnormalities remains unknown, which brings about an enormous burden for patients, families, and society. The spindle assembly checkpoint (SAC) controls the normal procedure of chromosome disjunction and may take part in the process.

**Objective:** The aim of this study was to explore the association between polymorphisms of *MAD1L1* rs1801368 and *MAD2L1* rs1283639804, involved in SAC and fetal chromosome abnormalities.

**Methods:** The case–control study collected 563 cases and 813 health controls to test the genotypes of *MAD1L1* rs1801368 and *MAD2L1* rs1283639804 polymorphisms by polymerase chain reaction–restrictive fragment length polymorphism methods (PCR-RFLP).

**Results:** *MAD1L1* rs1801368 polymorphism was associated with fetal chromosome abnormalities alone or combined to lower homocysteine (HCY) levels (alone: dominant: OR: 1.75, 95%CI: 1.19–2.57, and *p* = 0.005; CT vs. CC: OR = 0.73, 95%CI: 0.57–0.94, and *p* = 0.016; lower HCY: C vs. T: OR = 0.74, 95%CI: 0.57–0.95, and *p* = 0.02; dominant: OR = 1.75, 95%CI: 0.79–1.92, and *p* = 0.005). No significant differences were found in other genetic models or subgroups (*p* > 0.05, respectively). *MAD2L1* rs1283639804 polymorphism revealed a sole genotype in the studied population. HCY is significantly associated with fetal chromosome abnormalities in younger groups (OR: 1.78, 95%CI: 1.28–2.47, and *p* = 0.001).

**Conclusion:** The results implied that the polymorphism of *MAD1L1* rs1801368 may become the susceptibility factor to fetal chromosome abnormalities alone or combined to lower HCY levels but not to *MAD2L1* rs1283639804 polymorphism. In addition, HCY significantly affects fetal chromosomal abnormalities in younger women.

## Introduction

Fetal aneuploidy and chromosome structural abnormalities can lead to a spectrum of diseases, ranging from miscarriage, stillbirth, and other adverse pregnancy outcomes to adults with neurological disorders (e.g., copy number variant, CNV) (Shahine and Lathi, 2015; Yilmaz et al., 2017). Many maternal factors contributed to embryonic chromosomal aberrations, such as age, genetic polymorphism, reproductive history, and immune and endocrine dysfunction (Ozawa et al., 2019). It is reported that polymorphisms in folate-homocysteine pathway genes are related to chromosomal breaks and fetal chromosomal aneuploidy (Enciso et al., 2016). Environmental factors were also implicated in embryonic chromosomal aberrations, such as drug and pesticide exposure may increase the risk of embryonic DNA damage (Scheuerle and Aylsworth, 2016). Most embryos are aneuploidy derived from gamete cells, especially oocytes. However, the exact causes and etiopathogenetic mechanisms of teratogenesis are not well demonstrated (Baldacci et al., 2018; Toufaily et al., 2018).

The mitotic checkpoint system is a well-known major mechanism essential for maintaining the cell’s genomic stability. Consequently, an important monitoring system in cells has been found, named the mitotic spindle assembly checkpoint (SAC), which blocks the anaphase initiator stage until all chromatids correct their pairs (Bolanos-Garcia et al., 1969). Meiosis is considered to be a special form of mitosis, in which some mitotic cell cycle proteins play an important role and are crucial to the process of chromosomal disjunction (Avram et al., 2014). One noteworthy regulatory pathway with elevated roles in meiosis is the SAC. The mechanism of the SAC in meiosis has recently been discovered (Sun and Kim, 2012). The function of SAC protein components is similar in mitosis and meiosis. The weaker SAC signaling in human oocytes could lead to the high rate of aneuploidy compared to that in mice, according to kinetochore–microtubule attachment errors in human oocytes until anaphase I (Holubcova et al., 2015; Gruhn et al., 2019). MAD1L1 and MAD2L1 are important SAC protein components. MAD1L1 may contribute to metaphase delay during meiosis by restraining the activity of the anaphase-promoting complex/cyclosome (APC/C) to ensure proper alignment of the homologous chromosomes and sister chromatids. Initial functional and mutant investigations in different species elaborated that the loss of MAD1LI results in misaligned chromosomes, which proceed directly to anaphase I (Zhang et al., 2004; Stein et al., 2007).

MAD2L1 has been reported to play a more important role in meiosis than mitosis. Functional studies of MAD2L1 revealed that the gene regulates meiotic chromosome segregation and APC/C activity. The absence of MAD2L1 can cause the shortened duration of meiosis I, and meiotic spindle disorder, chromosome mispairing, and chromosome non-disjunction in meiosis I were observed in mouse oocytes (Sun and Kim, 2012). Furthermore, aneuploidy gametes can still be developed, although there are fewer cells with chromosome non-disjunction in meiosis I (Cairo et al., 2020).

Changes in MAD1L1 and MAD2L1 levels were detected in many cancer cell lines and tumor biopsy samples. Changes in MAD1L1 and MAD2L1 may serve as biomarkers for cancer as a potential strategy during anti-cancer treatment (Schuyler et al., 2012). The genotypic polymorphisms of *MAD1L1* and *MAD2L1* genes significantly increased the risk of certain cancers. The single nucleotide polymorphism C>T (rs1801368) transition in the *MAD1L1* gene nucleotide 1673 resulted in the transformation of arginine into histone in codon 558, leading to changes in the chromosomal disjunction domain of the MAD2L1-binding leucine zipper domain. This polymorphic change is associated with the chemotherapeutic response in advanced epithelial ovarian cancer patients (Santibanez et al., 2013). The effect of maternal *MAD2L1* Leu84Met polymorphism rs1283639804 G>A on fetal chromosome abnormalities is unclear.

HCY is considered an important intrinsic factor in fetal chromosomal abnormalities (James et al., 1999). We evaluated the previous paper and found that HCY was a significant risk factor for fetal chromosomal abnormalities. Whether HCY affects other biological events related to the formation and development of gametes or embryos is worth exploring due to its important role.

Given the important roles of MAD1L1 rs1801368 and MAD2L1 rs1283639804 in the SAC, we hypothesized that the two polymorphisms may disrupt the protein structure and function, which, in turn, may be associated with susceptibility to fetal chromosomal abnormalities. No studies have been conducted to assess the impact of MAD1L1 and MAD2L1 polymorphisms on fetal chromosomal abnormalities. We conducted this hospital-based case–control study, including two loci, aiming to explore the potential association between maternal genetic polymorphisms of *MAD1L1* rs1801368 and *MAD2L1* rs1283639804 genes with fetal chromosome abnormalities.

## Materials and methods

### Study subjects

The study recruited patients from the Department of Medical Genetics and included all cases and normal control pregnant women diagnosed with a fetal anomaly and normal chromosomal findings, respectively, First People’s Hospital in Yunnan Province, between January 2018 and April 2022 when they carried out an antenatal diagnosis in their second trimester. Ethics approval for this study was obtained from the institutional Ethics Committee (KHLL2020-KY025). Informed consent was signed by all registered participants. This study was aligned with the Strengthening the Reporting of Observational Studies in Epidemiology (STROBE) statement.

The sample size calculation for the case–control study was performed using the success sample size calculator. Based on the allele frequencies of the C allele, the frequency of *MAD1L1* rs1801368 in Chinese individuals is about 25%, reaching a confidence interval (CI) level of 95%, a minimum odds ratio (OR) of 1.5 for estimating the risk of a fetal chromosomal anomaly, and a margin of error of 5%, with a case–control ratio of 1:1.6 and with a case size of 500 pregnant women and 800 controls in the study; statistical power was achieved at the 95.37% level. Then, the sample collection was stopped when the size was reached.

The matching criteria for the case–control study were pregnant women, who made a prenatal diagnosis during the second trimester between 18 and 22+6 weeks’ gestation and tested for HCY concentration within 1 week. A pregnant woman determined to have a fetal chromosomal abnormality was enrolled as a case, and one or two age-matched pregnant women with HCY tested to date were also chosen as controls (2-year interval) from the same institute.

### Genotype determination

An Animal Genomic DNA Kit (Qingke Biotech Co., Ltd., China) was used to extract genomic DNA from peripheral blood samples. The polymerase chain reaction (PCR)-based restriction fragment length polymorphism (RFLP) was used to test all polymorphisms. *MAD1L1* rs1801368 located in exon 17 of MAD1L1 was amplified by PCR using the following primer pair: 5′-gtg​ggg​ggt​gcc​tac​ctg​cca​cct​cct​t-3'/5′-ggg​cca​tgg​tga​cct​gtg​ctg​tgt​gtg​tt-3′. The amplified 241-bp output was then digested by BsTU1 to determine the genotype. Amplification of MAD2L1 was carried out using the following primer: 5’ttg​ctg​agg​ata​ggg​agt​gg3′/5’ttc​ttt​tcc​ata​ggt​gac​tga​gg3′, and the PCR product fragments of 370 bp were digested by restriction endonuclease AlwN I (Guo et al., 2010). A random sample size of 20% was chosen to confirm the results of the restriction fragment length polymorphism assay by Sanger sequencing.

### HCY measurement

The level of HCY in the maternal plasma was measured by liquid chromatography coupled with a tandem mass spectrometry (LC-MS/MS) method (Waters ACQUITY TQ Detector). The LC-MS/MS system was equipped with a Waters 2795 liquid chromatograph (Waters 1525u Binary HPLC Pump) and an autosampler (Waters 2,777 sample manager) for the purpose of analysis. With reference to the manufacturer’s instructions for the use of the HCY kit and the standard operating procedure (SOP) of the National Center for Clinical Laboratories (NCCL) of China as a reference, after sample collection and pretreatment, the plasma HCY level was checked. The coefficient of variation was less than 5% for batches of HCY measurements.

### Statistical analysis

The statistical software package SPSS (version 17.0; SPSS, Inc., Chicago, IL, United States) was applied to analyze the data. Chi-squared tests between the characteristics were performed for qualitative variables, and Student’s t-test was performed for quantitative variables between cases and controls, respectively. The Hardy–Weinberg equilibrium was determined by Pearson’s chi-squared test for the case and control groups separately. We used binary logistic regression to calculate odds ratios (ORs) for the *MAD1L1* rs1801368 polymorphism between cases and controls. We further estimated the interactions between the *MAD1L1* rs1801368 polymorphism and maternal HCY level, with respect to fetal chromosome abnormalities. Based on controls, the median HCY value 6.76 μmol/L was determined and used for subject clustering. Statistical significance was accepted at a *p*-value of <0.05.

## Results

### Study samples

A total of 1,376 pregnant women were recruited for the study, comprising 563 cases and 813 normal controls. Table 1 shows the results for age, week of prenatal diagnosis of pregnancy, previous pregnancy times, delivery times, nationality, and prenatal diagnosis, and there was no significant difference between the two groups (*p* > 0.05, respectively). A total of 563 cases contain 209 fetal trisomy 21, 25 fetal trisomy 18, 17 fetal trisomy 13, 79 sex chromosome aneuploidies, 67 duplications, 82 deletions, 38 duplication/deletion, 30 inversions, and 16 translocation abnormalities. Of these, 1,125 subjects completed the HCY measure and were analyzed further. In the study, the genotype distribution in both groups agreed with the Hardy–Weinberg equilibrium (*p* > 0.05).

**TABLE 1:** Characteristics of study subjects for cases and controls.

Item	Control (n = 811)	Case (n = 563)	*p*-value
Age (years)	32.41 ± 5.99	31.76 ± 6.04	0.06
BMI			
Prenatal diagnosis pregnancy week (weeks)	20.27 ± 2.49	20.54 ± 2.76	0.14
Pregnancy times	2.77 ± 1.45	2.98 ± 1.51	0.05
Delivery times	0.71 ± 0.58	0.72 ± 0.64	0.66
Minorities			0.75
Han	623 (76.6%)	429 (76.1%)	0.65
Bai	72 (8.8%)	42 (7.4%)	
Yi	31 (3.8%)	25 (4.4%)	
Hui	19 (2.3%)	10 (1.7%)	
Dai	15 (1.8%)	13 (2.3%)	
Others	53 (6.5%)	44 (7.8%)	
Prenatal diagnosis results			
Trisomy 21		209 (63.3%)	
Trisomy 18		25 (7.5%)	
Trisomy 13		17 (5.1%)	
Sex chromosome aneuploidy		79 (23.9%)	
Duplication		67 (28.7%)	
Deletion		82 (35.1%)	
Insertion/duplication/deletion		38 (16.3%)	
Inversion		30 (12.8%)	
Translocation		16 (6.8%)	

### Association between *MAD1L1* rs1801368 and *MAD2L1* rs1283639804 G>A polymorphisms and fetal chromosomal instabilities

The *MAD2L1* rs1283639804 G>A polymorphism revealed only GG genotype in the population as a whole in the study. We just assessed only the association between the *MAD1L1* rs1801368 polymorphism and fetal chromosomal abnormalities, and a significant correlation was observed in the dominant and CT groups vs. the model for CC in Chinese females (OR: 1.35, 95%CI: 0.57–0.94, and *p* = 0.016) (listed in Table 2).

**TABLE 2:** Distribution of alleles, genotypes, and genetic models of the *MAD1L1* rs1801368 C>T polymorphism among cases and controls.

*MAD1L1* rs1801368 C>T		Control (n = 813)	Case (n = 563)	OR (95%CI)	*p*-value
Genotype	CC	201 (24.7%)	171 (30.4%)	ref	
	CT	425 (52.3%)	264 (46.9%)	0.73 (0.57–0.94)	0.016
	TT	187 (23.0%)	128 (22.7%)	0.81 (0.59–1.09)	0.16
Allele	C	827 (50.9%)	606 (53.8%)	ref	
	T	799 (49.1%)	520 (46.2%)	0.89 (0.76–1.04)	0.14
Dominant	CC vs. TT + CT			1.35 (1.06–1.72)	0.02
Recessive	CC + CT vs. TT			1.02 (0.79–1.31)	0.91
Hardy–Weinberg equilibrium		0.19	0.18		

OR was adjusted by age, and HCY, value.

#### The interaction of *MAD1L1* rs1801368 and HCY levels for fetal chromosomal instability

Participants were separated into two groups based on the cut-off value for the median HCY level based on the control population. Stratification analysis was then performed, and the results are listed in Table 3. In the subgroup of fetal chromosome abnormalities with lower HCY levels, under the dominant model, the CC genotype frequency was significantly higher than the control (OR: 1.75, 95%CI: 1.19–2.57, and *p* = 0.005). Moreover, the CT genotype was negatively associated with fetal chromosome abnormalities compared to the CC genotype (OR = 0.73, 95%CI: 0.57–0.94, and *p* = 0.016). The C allele was found to be significantly elevated in cases compared to controls (OR = 0.74, 95%CI: 0.57–0.95, and *p* = 0.02), and there is a significant difference between cases and controls in the presence of the dominant model (OR = 1.75, 95%CI:0.79–1.92, and *p* = 0.005). However, there were no significant differences in the other genetic models and in the higher HCY group in all of the genetic models (*p* > 0.05, respectively).

**TABLE 3:** HCY stratification analysis of the *MAD1L1* rs1801368 polymorphism between case and control groups.

*MAD1L1* rs1801368 C>T			Control (n %)		Case (n %)		OR (95%CI)	*p*-value
Lower HCY	Genotype	CC	122	23.90%	57	35.80%	References	
		CT	271	53.00%	71	44.70%	1.01 (0.96–1.05)	0.73
		TT	118	23.10%	31	19.50%	0.99 (0.93–1.06)	0.79
	Allele	C	515	50.40%	185	58.20%	References	
		T	507	49.60%	133	41.80%	0.74 (0.57–0.95)	0.02
	Dominant	CC vs. TT + CT					1.75 (1.19–2.57)	0.005
	Recessive	CC + CT vs. TT					1.23 (0.79–1.92)	0.35
Higher HCY	Genotype	CC	78	26.00%	47	30.30%	References	
		CT	154	51.30%	62	40.00%	1.02 (0.95–1.08)	0.34
		TT	68	22.70%	46	29.70%	0.96 (0.91–1.02)	0.15
	Allele	C	310	51.70%	156	50.30%	References	
		T	290	48.30%	154	49.70%	1.05 (0.80–1.39)	0.71
	Dominant	CC vs. TT + CT					1.24 (0.81–1.91)	0.32
	Recessive	CC + CT vs. TT					0.70 (0.45–1.08)	0.10

OR was adjusted by age.

#### HCY stratification analysis between different age groups for the fetal chromosomal instability

Participants were separated into two groups based on the cut-off value for the median HCY level based on the control population and the age of 35 years old. Stratification analysis was carried out, and the results are listed in Table 4. In the younger group (<35 years old) and total population, HCY significantly raised the risk of fetal chromosomal abnormalities (OR = 1.78, 95%CI = 1.28–2.47, and *p* = 0.001; OR = 165, 95%CI = 1.27–2.15, and *p* = 0.001, respectively). However, in the advanced group, no significant difference was found (OR = 1.44, 95%CI = 0.92–2.25, and *p* = 0.011).

**TABLE 4:** HCY stratification analysis in different age groups between case and control groups.

Age group	Lower HCY		Higher HCY		OR (95%CI)	*p*-value
	(<6.76 μmol/L)		(≥6.76 μmol/L)			
	Control (n %)	Case (n %)	Control (n %)	Case (n %)		
<35 years old	332 (77.2%)	98 (22.8%)	200 (65.6%)	105 (34.4%)	1.78 (1.28–2.47)	0.001
≥35 years old	179 (74.6%)	61 (25.4%)	100 (67.1%)	49 (32.9%)	1.44 (0.92–2.25)	0.11
Total	511 (76.3%)	159 (23.7%)	300 (66.1%)	154 (33.9%)	1.65 (1.27–2.15)	0.001

## Discussion

In eukaryotic cells, MAD1 controls chromosome segregation *via* the SAC mechanism. In mitosis, the cell cycle arrest was impaired when MAD1 expression was downregulated by treatment with microtubule stabilizing and microtubule destabilizing agents (Kienitz et al., 2005). Mutation frequencies of the SAC genes remained low, and only rare polymorphisms in *MAD1L1*, *MAD2L1*, and *BUB1b* were found in certain cancers involved in the modulation of the cell cycle arrest (Su et al., 2016; Bandala-Jacques et al., 2020; Sun et al., 2020).

We conducted a case–control study to explore the potential association of maternal *MAD1L1* rs1801368 and *MAD2L1* rs1283639804 polymorphisms with a chromosomal abnormality in the fetus. It is worth noting that *MAD2L1* rs1283639804 G>A loci in the southwestern Chinese women’s population revealed only a single genotype in our study, suggesting that this polymorphism is conserved in the studied population and the limitation to the small sample size presented for our study. This is a low minor allele frequency (MAF) based on the NCBI database [A = 0.000004/1 (GnomA Dexomes)], but some investigators have found a polymorphism of *MAD2L1* rs1283639804 in combination with the polymorphism of *MAD1L1* rs1801368 conferring colorectal and lung cancers (Guo et al., 2010; Zhong et al., 2015). Hence, we preliminarily explored the possible association of the polymorphism with fetal chromosome abnormalities. The final result suggested, perhaps, some other polymorphisms in *MAD2L1* but not rs1283639804 related to fetal chromosome abnormalities, which need to be further studied.

We then found a significant difference in the frequency for *MAD1L*1 rs1801368 C>T between cases and controls. In some studies, the polymorphism in the *MAD1L1* rs1801368 T allele has been found to be associated with certain kinds of cancer that showed somatic cell aneuploidy (Santibanez et al., 2013; Zhong et al., 2015; Bandala-Jacques et al., 2020). However, interestingly, our result of *MAD1L1* rs1801368 C>T was contrary to those reports. We have followed possible explanations that 1) the process of regulating meiosis may be different from mitosis and is a variation of different sorts of species; 2) *MAD1L*1 rs1801368 is only a very small factor in comparison to the entire SAC array; 3) particularly, when the interactions of gene–gene, gene–environment, or other impact factors are jointly involved (Alamgir et al., 2022), certain environmental factors may play a crucial role in the event of gamete meiosis and subsequent embryo development; and 4) Differences could exist due to genetic heterogeneity in different populations (Baker et al., 2007; Karban et al., 2016).

We also performed an additional analysis combining the *MAD1L1* rs1801368 C>T polymorphism with levels of maternal HCY, which is a major related factor for fetal chromosome abnormalities described in our previous article. The lower HCY subgroup [HCY <6.755 μmol/L (median value)] had a significantly higher frequency of both the CC genotype and the C allele in the cases compared to the control (dominant model: OR = 0.74, 95%CI: 0.57–0.95, and *p* = 0.02; C vs. T allele: OR = 1.75, 95%CI: 0.79–1.92, and *p* = 0.005), which did not occur in the higher HCY group (*p* > 0.05, respectively). Suggesting that *MAD1L1* rs1801368 likely incorporates physiological environmental factors that affect the biological course of the event, even though *MAD1L*1 has not been included in the circulation of folate-acid homocysteine, it is well known that methylation modification has been controlled by circulating folate-acid homocysteine and affects the expression of the genes involved (Ma et al., 2022; Wang et al., 2022). However, the mechanism of how *MAD1L*1 methylation interacts with the level of HCY in the internal environment needs to be explored. Alternatively, the outcome of most fetuses with chromosomal abnormalities was miscarriage during early pregnancy, and over 50% of the tissue from miscarriages was detected as chromosomal aneuploidy, and only a small portion can survive. These issues need to be elucidated if the role of the SAC is to either initiate or maintain the continued development of gametes and chromosomal abnormality embryos.

HCY was an important biomarker in the pathway of folate acid and HCY, which was regarded as a risk factor of pregnancy outcomes, such as neural tube defects, Down’s syndrome, and some adverse pregnancy outcomes (Straaten et al., 2001; Liu et al., 2017). It is well known that age was an independent risk of fetal chromosomal abnormalities. In our case–control study matched with age, the result revealed that HCY was a risk factor of fetal chromosomal abnormalities for the younger pregnant women but not for the advanced pregnant women; perhaps the age was the main risk factor of fetal chromosome abnormalities.

The study is limited in some ways. 1) Some selection bias is introduced by the subjects selected from the prenatal diagnosis group. 2) Stratification analysis according to fetal numerical and structural abnormalities was not performed for the small sample size. 3) Of concern were only two *MAD1L1* and *MAD2L1* polymorphisms in the genes controlling SACs, and it is necessary to investigate other polymorphisms or genes such as *Mad3*/*BubR1*, *Bub1*, *Bub3*, and *Mps11*. 4) The association was in the opposite direction to the other reports, and its function during the mitotic process may lack a rational explanation. Depending on the meiosis complex, the cause of fetal chromosomal abnormalities requires further investigation. 5) HCY levels were tested during their second trimester, missing the value of pre-conception and early pregnancy HCY and potentially missing the best time frame that reflects the influence of HCY on fetal chromosomal stabilities. 6) The case group in this study also included some *de novo* chromosomal structural abnormalities, and origination was determined neither by the mother nor by the father.

In spite of these limitations, this study has some strengths. This study is the first to access the association between maternal polymorphisms implicated in SAC and fetus’ chromosomal abnormalities. In addition, the results then highlight the point that perhaps *MAD1L1* rs1801368 C>T interacts with the level of internal HCY to confer susceptibility to fetal chromosomal abnormalities. All participants had an explicit fetal diagnosis with no confounders.

In conclusion, our study initially found the potential association of the maternal *MAD1L1* rs1801368 C>T polymorphism with fetal chromosomal abnormalities. If lower levels of HCY are concerned, CC and C alleles may be linked to the higher risk of fetal chromosomal abnormalities. Additional studies were needed to clarify the genetic effect of the *MAD1L1* rs1801368 C>T polymorphism on human chromosomal instability gametes or the occurrence of embryos and ongoing development.

## Data Availability

The original contributions presented in the study are included in the article/Supplementary Material; further inquiries can be directed to the corresponding author.
